# Persistent Luminescence Zn_2_GeO_4_:Mn^2+^ Nanoparticles Functionalized with Polyacrylic Acid: One-Pot Synthesis and Biosensing Applications

**DOI:** 10.1021/acsami.2c21735

**Published:** 2023-03-27

**Authors:** Roxana M. Calderón-Olvera, Encarnación Arroyo, Aaron M. Jankelow, Rashid Bashir, Enrique Valera, Manuel Ocaña, Ana Isabel Becerro

**Affiliations:** † Instituto de Ciencia de Materiales de Sevilla (CSIC-US), c/Américo Vespucio, 49, Seville 41092, Spain; ‡ Department of Bioengineering, University of Illinois at Urbana-Champaign, Urbana, Illinois 61801, United States; § Nick Holonyak Jr Micro and Nanotechnology Lab, University of Illinois at Urbana-Champaign, Urbana, Illinois 61801, United States; ∥ Department of Electrical and Computer Engineering, University of Illinois at Urbana-Champaign, Urbana, Illinois 61801, United States; ⊥ Department of Mechanical Science and Engineering, University of Illinois at Urbana-Champaign, Urbana, Illinois 61801, United States; # Center for Genomic Diagnostics, Woese Institute for Genomic Biology, Urbana, Illinois 61801, United States

**Keywords:** persistent luminescence, nanoparticles, zinc germanate, functionalization, colloidal stability, photostability, biosensing, immunoassay

## Abstract

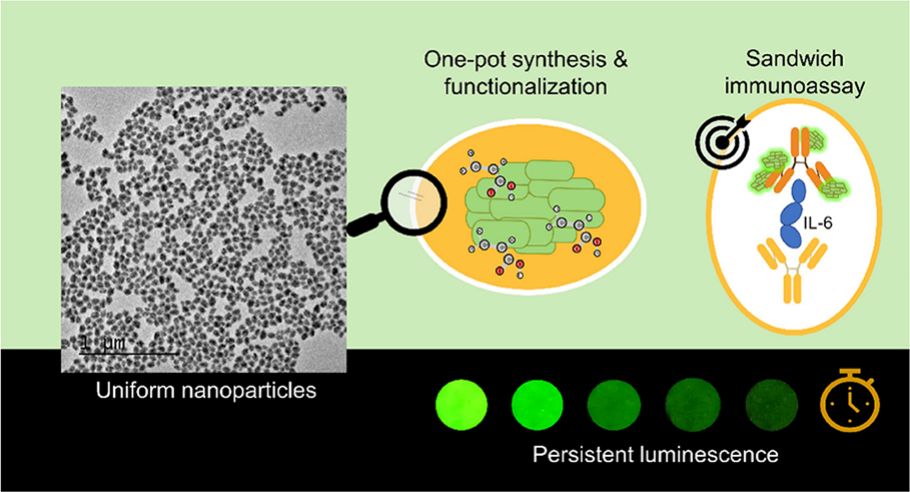

Zinc germanate doped with Mn^2+^ (Zn_2_GeO_4_:Mn^2+^) is known to be a persistent luminescence green phosphor with potential applications in biosensing and bioimaging. Such applications demand nanoparticulated phosphors with a uniform shape and size, good dispersibility in aqueous media, high chemical stability, and surface-functionalization. These characteristics could be major bottlenecks and hence limit their practical applications. This work describes a one-pot, microwave-assisted hydrothermal method to synthesize highly uniform Zn_2_GeO_4_:Mn^2+^ nanoparticles (NPs) using polyacrylic acid (PAA) as an additive. A thorough characterization of the NPs showed that the PAA molecules were essential to realizing uniform NPs as they were responsible for the ordered aggregation of their building blocks. In addition, PAA remained attached to the NPs surface, which conferred high colloidal stability to the NPs through electrostatic and steric interactions, and provided carboxylate groups that can act as anchor sites for the eventual conjugation of biomolecules to the surface. In addition, it was demonstrated that the as-synthesized NPs were chemically stable for, at least, 1 week in phosphate buffer saline (pH range = 6.0–7.4). The luminescence properties of Zn_2_GeO_4_ NPs doped with different contents of Mn^2+^ (0.25–3.00 mol %) were evaluated to find the optimum doping level for the highest photoluminescence (2.50% Mn) and the longest persistent luminescence (0.50% Mn). The NPs with the best persistent luminescence properties were photostable for at least 1 week. Finally, taking advantage of such properties and the presence of surface carboxylate groups, the Zn_2_GeO_4_:0.50%Mn^2+^ sample was successfully used to develop a persistent luminescence-based sandwich immunoassay for the autofluorescence-free detection of interleukin-6 in undiluted human serum and undiluted human plasma samples. This study demonstrates that our persistent Mn-doped Zn_2_GeO_4_ nanophosphors are ideal candidates for biosensing applications.

## 1 Introduction

Persistent luminescence nanoparticles (PLNPs) are a kind of nanomaterial that presents afterglow for minutes to hours after the excitation radiation has stopped.^1^ The fact that PLNPs do not need excitation during the luminescence detection period is very advantageous when these PLNPs are used as nanoprobes for biosensing as they allow for the elimination of background autofluorescence and light scattering interference associated with biological species.^2−7^ In addition to good afterglow properties, PLNPs used in biosensing applications must be well-dispersed and present a regular shape and uniform size and high colloidal and chemical stability.^2^ Moreover, functionalization of their surface is highly desirable to provide anchoring sites for the probe molecules to be used in particular bioassay applications.^6^

Among PLNPs, those based on Zn_2_GeO_4_:Mn^2+^ have been proposed as good candidates for biosensing as this material was found to show a green luminescence, after UV excitation, that can be detected 3 h after the excitation has been stopped.^8,9^ Zn_2_GeO_4_ is isostructural with phenacite (Be_2_SiO_4_). It crystallizes in the rhombohedral system and consists of Ge and Zn tetrahedra alternating in a pattern running parallel to the *c* axis. The structure has both four- and six-membered rings in the plane perpendicular to the *c* axis and three-membered rings in planes parallel to the *a* and *b* axes.^10^ According to the classic study by Hartman and Perdok about the relations between structure and morphology of crystals,^11^ the morphology of a crystal is governed by chains of strong bonds running through the structure. Such chains in the phenacite Zn_2_GeO_4_ structure correspond to Ge and Zn tetrahedral chains parallel to the *c* axis. Therefore, it is expected that Zn_2_GeO_4_ crystals prefer to grow in the direction of the *c* axis, giving rise to rod-like crystals.

Zn_2_GeO_4_:Mn^2+^ rod-like NPs with large aspect ratios are indeed reported in the vast majority of studies about PLNPs of this material. Such studies reported Zn_2_GeO_4_:Mn^2+^ rods obtained by hydrothermal reaction of zinc and manganese nitrates and germanium oxide in alkaline conditions in the absence of any additive, where the dimensions of the rods are determined by the temperature, time, and pH of the reaction.^12−20^ However, although it is essential that the NPs to be used in biomedicine are monodisperse in nature and exhibit colloidal and chemical stability in physiological medium, these physicochemical features have never been demonstrated for the reported Zn_2_GeO_4_:Mn^2+^ nanorods by suitable methods such as dynamic light scattering (DLS) and ion coupling plasma (ICP).^21^ In addition, despite the importance of surface functionalization for bioapplications, only a few studies report surface-functionalized Zn_2_GeO_4_:Mn^2+^ NPs, all of which require a second-stage functionalization reaction following the hydrothermal synthesis.^12−14,20^

In this study, uniform Zn_2_GeO_4_:Mn^2+^ NPs of ellipsoidal shape and approximate dimensions 57 nm × 80 nm, functionalized with carboxylate groups, were synthesized following a simple one-pot method using polyacrylic acid (PAA) as the synthesis additive. The resulting PAA-functionalized NPs were well-dispersed in water and in a physiological-like medium and presented excellent colloidal and chemical stability in the latter, as inferred from DLS and ICP measurements. The NPs showed a strong green luminescence under ultraviolet (UV) light that persisted long after the excitation was stopped. These properties were optimized by systematically varying the Mn^2+^ doping level. Therefore, the PLNPs fulfilled the essential requisites to be used as luminescent nanoprobes in biomedicine. As a proof of concept, a generic sandwich immunoassay based on persistent luminescence was fabricated to prove the efficiency of the PAA-functionalized Zn_2_GeO_4_:Mn^2+^ NPs as transducers to detect interleukin-6 (IL-6) in undiluted human serum and undiluted human plasma samples. The presence of PAA on the surface of the NPs was essential as it provides carboxylate groups for further conjugation with the detection antibodies involved in the immunoassay. IL-6 is a proinflammatory cytokine secreted by immune cells whose presence in serum is a clear sign of infection.^22^ IL-6 is also expressed in blood during or after kidney disease^23^ and lung fibrosis,^24^ and even serious cases of SARS-CoV-2 have been recently correlated with high concentrations of IL-6 in serum samples.^25,26^

## 2 Experimental Section

### 2.1 NP Synthesis and Characterization

#### 2.1.1 Materials

The precursors used to synthesize the NPs were zinc acetate (Zn(CH_3_CO_2_)_2_, Sigma-Aldrich, 99.99%), germanium(IV) oxide (GeO_2_, Sigma-Aldrich, ≥99.99%), manganese(II) acetate tetrahydrate (Mn(CH_3_COO)_2_· 4H_2_O, Sigma-Aldrich, 99.99%), and poly(acrylic) acid (PAA, average Mw ∼ 1800, Sigma-Aldrich). Sodium hydroxide (NaOH, Sigma-Aldrich, ≥98%) was used to adjust the pH of the reaction media. Milli-Q water was used as the solvent and phosphate-buffered saline (PBS, Sigma Aldrich, pH = 7.4) as the medium to disperse the NPs for chemical and colloidal stability studies.

#### 2.1.2 NPs Synthesis

Persistent Zn_2_GeO_4_:Mn^2+^ NPs were synthesized through a hydrothermal method assisted by a microwave oven (MW) according to the following procedure: GeO_2_ (0.01 M) was dissolved in 5 mL of Milli-Q water adjusting the pH to 10.0 dropwise with NaOH (1 M). In a second vial, Zn(CH_3_COO)_2_ (0.02 M) was dissolved in 5 mL of Milli-Q water, stirring vigorously for 60 min. Next, Mn(CH_3_COO)_2_·4H_2_O (nominal contents 0.25, 0.50, 1.00, 2.00, 2.50, and 3.00 mol % referred to Ge) was incorporated into the Zn(CH_3_COO)_2_ solution and left under stirring for 20 min. Subsequently, PAA (2 mg·mL^–1^) was added and stirred for 40 min. Once this time had elapsed, the GeO_2_ solution was incorporated under stirring into the previous solution, and the pH of the final mixture was adjusted to 10.0 with NaOH (1 M). The resulting solution was immediately transferred to a 30 mL glass vial, placed in a microwave oven (Monowave 300, Anton Paar), and heated at 220 °C for 1 h. The resulting suspension was cooled down to room temperature and washed four times with distilled water in a centrifuge (Sorvall Legend X1R-Thermo Scientific) at 14000 rpm for 20 min. Finally, the so-purified particles were re-dispersed in Milli-Q water and dried at 50 °C for further analyses.

#### 2.1.3 NPs Characterization Techniques

The morphology of the Zn_2_GeO_4_:Mn^2+^ NPs was examined by transmission electron microscopy (TEM, JEOL2100Plus, 200 kV). To determine the nanoparticle size, about one hundred particles were measured from the TEM micrographs using the free ImageJ software. The crystalline structure was identified from X-ray powder diffraction (XRD) patterns recorded in a Panalytical X′PERT PRO equipped with an X-Celerator detector; the step size was set at 0.03° (2θ) and the counting time at 1000 s. Dynamic light scattering (DLS) measurements were performed in aqueous and PBS suspensions of the NPs (0.25 mg·mL^–1^) using a Malvern Zetasizer Nano-ZS90 that generated an intensity particle size distribution (PSD). The cumulants analysis of the intensity PSD gave two values, a mean value for the size (*z*-average size) and a width parameter known as the polydispersity index (PdI). ICP measurements were carried out using an iCAP 7200 ICP-OES Duo equipment. Concentrated hydrochloric acid (3 mL) and nitric acid (3 mL) were added to the NP powders (20 mg) and heated in a microwave oven at 230 °C to digest the samples prior to the ICP analysis. Fourier transform infrared (FTIR) spectra of the NPs diluted in KBr were recorded in a JASCO FT/IR Fourier transform spectrometer. Thermogravimetry (TG) curves were measured using a Q600 TA instrument, with a heating rate of 10 °C·min^–1^ in an air atmosphere.

Luminescence measurements (excitation and emission spectra) were carried out in an Edinburgh FLS100 spectrofluorometer. The wavelengths for excitation and emission were 290 and 535 nm, respectively. An aqueous suspension with a concentration of 1 mg·mL^–1^ was prepared for each Mn^2+^-doped sample for an accurate comparison of their luminescence properties. Persistent luminescence decay curves were measured using the above equipment. The detector was set at 535 nm, and the samples were excited at 290 nm for 5 min before recording the persistent luminescence decay curves. Digital photos of the Zn_2_GeO_4_:0.50%Mn^2+^ powder sample at different time intervals were taken after being irradiated with a UV (312 nm) lamp for 5 min using the following series of parameters: ISO: 3200, Integral: 1/16 s, EV: 0, and WB.

The colloidal stability of the synthesized NPs was evaluated at pH = 6.0 and pH = 7.4. With this purpose, a suspension consisting of 4 mL of Zn_2_GeO_4_:0.50%Mn^2+^ NPs dispersed in PBS (0.25 mg·mL^–1^) at the desired pH was kept undisturbed in a quartz cuvette at room temperature. The intensity PSD was recorded periodically by DLS and compared with the one obtained from the freshly prepared sample measured initially.

The chemical stability of the Zn_2_GeO_4_:0.50%Mn^2+^ NPs at pH = 6.0 and pH = 7.4 using PBS as the dispersing medium was assessed through TEM and ICP analyses. The PBS suspensions consisting of 2 mL of Zn_2_GeO_4_:0.50%Mn^2+^ NPs (1 mg·mL^–1^) were left under stirring at room temperature for several periods of time. The suspensions were then centrifuged, and the supernatants were collected for ICP analysis. The precipitates obtained after centrifugation were washed with distilled water and observed under the TEM for their comparison with the TEM micrographs of the freshly prepared sample.

Photostability of the Zn_2_GeO_4_:0.50%Mn^2+^ NPs in PBS suspension (1 mg·mL^–1^) at pH = 6.0 and pH = 7.4 was tested through the periodic measurement of their emission spectrum and persistent luminescence decay curve for 1 week.

### 2.2 ELISA Sandwich Immunoassay for IL-6 Detection Using PLNPs-Ab_*d*_ Probes

#### 2.2.1 Chemicals, Reagents, and Buffer Solutions

N-Hydroxylsulfoccinimide sodium salt (NHS, Cat# 56485-1G), Bradford Reagent (Cat# B6916-500 ML), and bovine serum albumin (BSA, Cat# 05479-10G) were purchased from Merck Sigma-Aldrich. 1-(3-(Dimethylaminopropyl)-3-ethyl carbodiimide hydrochloride (EDC, Cat# AC171440010) was purchased from Fisher Scientific. IL-6 monoclonal antibodies (MQ2-39C3, Cat# 14-7068-81; and MQ2-13A5, Cat# 14-7069-81), Human IL-6 Recombinant Protein (Cat# BMS341), Immuno Breakable Modules in White, Maxisorp 96-well plate (Cat# 463201), and Goat anti-Mouse IgG Fc Secondary antibody (Cat# SA5-10275) were purchased from Thermo Fisher Scientific.

Gibco PBS, pH = 7.4 (Cat# 11593377) was purchased from Fisher Scientific. PBS buffer was filtered with 0.1 μm membrane filters (Durapore), and the pH was adjusted to 6 (adding HCl 1 M) for the conjugation of PLNPs to detection antibodies. ELISA carbonate coating buffer (Cat# CB01100) and Pierce 20X TBS-Tween 20 Buffer (PBS-T, Cat# 28360) eBioscience were purchased from Thermo Fisher Scientific. Plasma from a human (P9523-5ML) and human serum (from human male AB plasma, USA origin, sterile-filtered, H4522-20ML) were purchased from Sigma-Aldrich and used in the sandwich immunoassay.

#### 2.2.2 Instrumentation

Excitation of samples was performed in a black box using a UV lamp (312 nm, Vilber Lourmat). Persistent luminescence decays were measured using a reader plate (Varioskan Lux, Thermo Fisher Scientific) in luminescence mode. The efficiency of the coupling strategy by the Bradford test was measured using the same equipment in absorbance mode. The unpaired t-test of the collected data was performed using GraphPad Prism 9.

#### 2.2.3 Conjugation of the Zn_2_GeO_4_:0.50%Mn^2+^ PLNPs to Detection Antibodies (PLNPs-Ab_*d*_ Probes)

Zn_2_GeO_4_:0.50%Mn^2+^ PLNPs were conjugated to detection antibodies (Ab_*d*_) by carbodiimide coupling chemistry^27^ using the following protocol: The PLNPs (11.5 mg), whose surface had been functionalized with PAA during their one-pot synthesis, were washed twice with PBS (pH = 6) by centrifugation in an Epperdorf MiniSpin centrifuge (13200 rpm, 3 min, 20 °C) in a 1.7 mL tube. After the last washing, the PLNPs were resuspended in PBS (90 μL, pH = 6) and mixed with EDC/NHS (10 μL, 200 mM/100 mM, in PBS pH = 6). The resulting mixture was stirred (850 rpm, 20 min, room temperature) to activate the carboxylic groups. Thereafter, the PLNPs were washed three times to remove the excess reagents. Once the last supernatant was discarded, the activated PLNPs were resuspended in PBS (100 μL, pH = 6), and the IL-6 monoclonal antibodies (MQ2-39C3, 50 μL, 500 μg·mL^–1^) were added to the suspension. The resulting PLNPs-Ab_*d*_ suspension was homogenized in a vortex (3 h, 850 rpm). Finally, the PLNPs-Ab_*d*_ were washed twice (13200 rpm, 3 min, 20 °C) with PBS (pH = 7.4) to remove the unbound antibodies. In these steps, supernatants were collected to quantify the unconjugated antibodies. The Bradford test^28^ and IgG antibodies were then used to examine the coupling strategy’s efficiency. The estimated conjugation efficiency was (81 ± 10) % (*n* = 7). Finally, the conjugated PLNPs to Ab_*d*_ were resuspended in 100 μL of PBS (filtered, pH = 7.4) obtaining a final concentration of 202.5 μg·mL^–1^ of Ab_*d*_. This suspension was stored at 4 °C until use.

#### 2.2.4 Sandwich Immunoassay Protocol

**Scheme 1:**
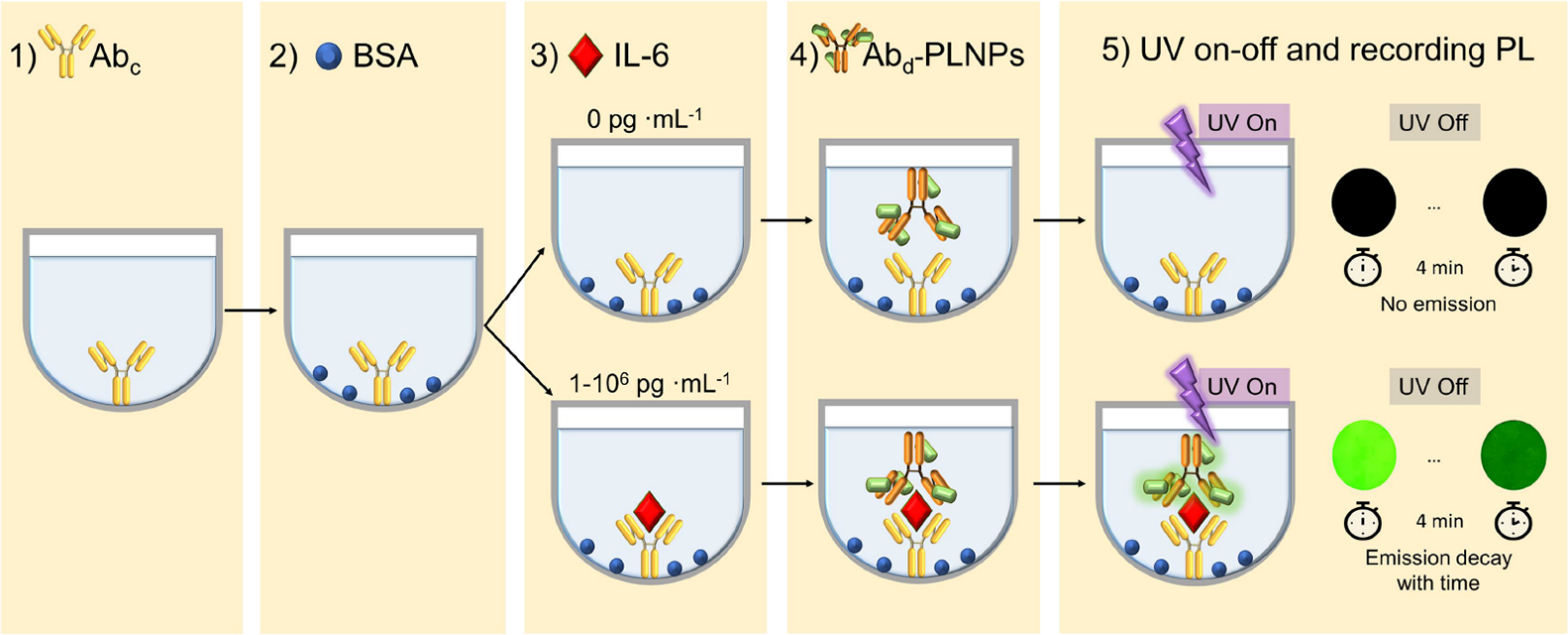
IL-6 Sandwich Immunoassay Procedure Based on PLNPs

The immunoassay protocol is schematized in Scheme 1 and consists of the following steps: (1) IL-6 monoclonal antibodies (MQ2-13A5) were used as capture antibodies (Ab_*c*_). The Ab_*c*_ (10 μg·mL^–1^ in carbonate coating buffer, 150 μL/well) were added to a MaxiSorp 96-well plate and incubated overnight (4 °C). (2) After the Ab_*c*_ were anchored to the plates, BSA (150 μL/well, 0.1% in PBS, pH = 7.4) was added (as a blocking agent) and incubated at 37 °C (90 min); (3) A range of concentrations of IL-6 (0–10^6^ pg·mL^–1^) was added to the plate (100 μL/well) and incubated at 37 °C (2 h). IL-6 was spiked to undiluted human serum, undiluted human plasma, and PBS (pH = 7.4) to perform three sandwich immunoassays; (4) the PLNPs-Ab_*d*_ nanoprobes (1.75 μL in PBS pH = 7.4, 150 μL/well) were added to the well plate and incubated at 37 °C (90 min). After steps 1–4, the wells were washed (3–5 times, in 1X PBS-T buffer). After step 4, PBS, pH = 7.4 (100 μL) was added to each well. (5) Each well was individually illuminated with a UV lamp (312 nm, 150 s) in a black box (the rest of the wells were covered with black paper); the well plate was then introduced in a Varioskan Lux equipment and the persistent luminescence decay of that well was recorded for 4 min (luminescence mode, integration time = 2 s). From the moment the lamp was switched off to the start of the persistence recording 40 s elapsed. Step 5 was repeated for each IL-6 concentration with 6 replicates for each one (*n* = 6). The integrated area of the persistent luminescence decays was normalized (by subtracting the integrated area of the control sample) and plotted versus IL-6 concentration.

## 3 Results

### 3.1 Synthesis, Morphology, Crystal Structure, and Surface Composition of the Zn_2_GeO_4_ NPs

**Figure 1:**
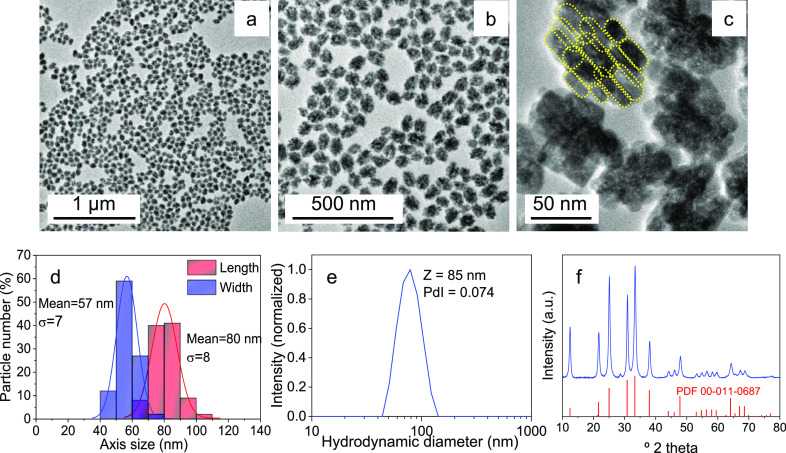
(a–c) TEM micrographs at different magnifications of the Zn_2_GeO_4_ NPs synthesized by aging (220 °C for 1 h in a microwave oven) an aqueous solution containing GeO_2_ (0.01 M), Zn(OAc)_2_ (0.02 M), and 2 mg·mL^–1^ PAA (Mw = 1800). The yellow dashed lines in the highest magnification image are guides to the eye to appreciate the oriented aggregation of the building blocks. (d–f) Particle size histogram, intensity PSD in Milli-Q water (obtained from DLS), and XRD pattern, respectively, of the Zn_2_GeO_4_ particles shown above.

For the sake of simplicity, we have first addressed the synthesis of the undoped material and then applied the experimental parameters found to synthesize the Mn^2+^-doped NPs. Figure 1a–c shows different-magnification TEM micrographs of the NPs obtained after aging, at 220 °C for 1 h in a microwave oven, an aqueous solution containing GeO_2_ (0.01 M), Zn(OAc)_2_ (0.02 M), and 2 mg·mL^–1^ PAA (Mw = 1800). The NPs presented an ellipsoid shape, the short and long axis dimensions being 57 nm (σ = 7) and 80 nm (σ = 8), respectively (Figure 1d). The *z*-average size obtained from the DLS measurement of the NPs suspended in distilled water (pH = 6, Figure 1e) was 85 nm, which is very close to the mean length of the NPs measured in TEM micrographs. This result indicates a very good dispersibility of the NPs in water. Likewise, the PdI value obtained from DLS was lower than 0.1, which indicates a reasonably narrow monomodal sample.^29^

Interestingly, increasing the amount of PAA to 3 mg·mL^–1^ resulted in aggregated NPs with the same size and morphology as those shown in Figure 1 while decreasing it to 1 mg·mL^–1^ gave rise to well-dispersed NPs but of larger size (∼180 nm × ∼100 nm) (Figure S1). Therefore, we selected the NPs synthesized in the presence of 2 mg·mL^–1^ PAA for all studies presented from now on.

**Figure 2:**
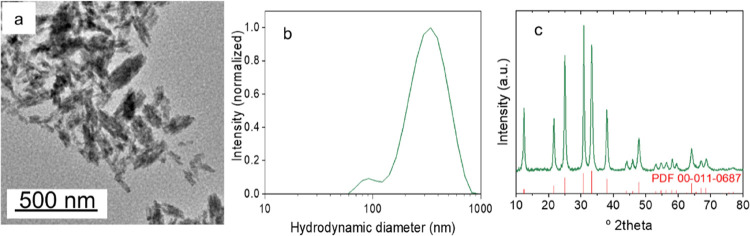
TEM image (a), intensity PSD obtained from DLS (in Milli-Q water) (b), and XRD pattern (c) corresponding to Zn_2_GeO_4_ particles obtained in the same experimental conditions as those shown in Figure 1 but in the absence of PAA.

The powder XRD pattern of the NPs (Figure 1f) was compatible with the crystallization of rhombohedral Zn_2_GeO_4_ (phenakite structure) as shown by the good match between the experimental reflections and the Powder Diffraction File (PDF) 00-011-0687^30^ corresponding to such phase. The crystallite size, calculated using the Scherrer equation from the width at half maximum of the reflection at 33.4 °2 theta, was ∼17 nm. This size, clearly smaller than the NPs’ dimensions, indicated that the NPs were polycrystalline in character, i.e., they were formed by the ordered aggregation of smaller subunits. In fact, the high-magnification images in Figure 1b,c allow the observation of different building blocks (primary particles) forming a single NP. Such a formation mechanism has been observed for other monodisperse colloidal particles,^31,32^ and different models have been developed to explain the mean size and size distribution of the final particles.^33^ The models assume that primary particles are formed through the classical nucleation and growth theory and that the aggregation process requires a proper balance between the attractive van der Waals forces and the repulsive (electrostatic and steric) forces acting in a colloidal system. The attractive forces are determined by the solid composition while the repulsive forces depend on temperature, ionic strength, precursors concentration, number of primary particles, and the presence of additives (polymers, ligands, or surfactants).^33^ In our case, the use of PAA as a synthesis additive was crucial for the precipitation of uniform NPs as the product obtained in the absence of PAA, keeping the rest of the experimental conditions unchanged, consisted of fully heterogeneous, larger aggregates and some isolated nanorods (Figure 2a). The aqueous suspension of such a precipitate presented a *z*-average size of 295 nm (Figure 2b), well above the nanometer range. The XRD pattern of the dried precipitate (Figure 2c) was compatible with the crystallization of rhombohedral Zn_2_GeO_4_, as observed for the NPs synthesized in the presence of PAA, although in this case, the crystallite size was slightly larger (∼20 nm). Therefore, the PAA molecules in the hydrothermal reaction limited somehow the growth of the building blocks and favored their oriented aggregation, thus allowing the formation of NPs with a homogeneous size and uniform shape.

**Figure 3:**
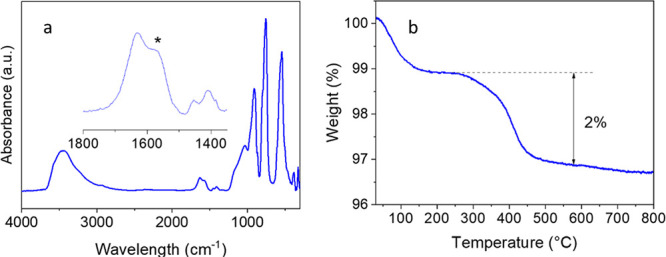
FTIR spectrum (a) and TG curve (b) of the Zn_2_GeO_4_ nanoparticles shown in Figure 1.

FTIR spectroscopy and TG analyses gave a deeper insight into this behavior. The FTIR spectrum of the sample synthesized in the presence of PAA (Figure 3a) showed the bands expected from the adsorbed water (at 3400 and 1632 cm^–1^) and the Zn_2_GeO_4_ crystal structure (below 1200 cm^–1^).^34^ In addition, a set of features were observed in between 1400 and 1560 cm^–1^, compatible with the presence of carboxylate groups on the surface of the NPs^35^ that must come from the PAA molecules used as the synthesis additive. The TG curve obtained for this sample (Figure 3b) agreed well with such assignment as it showed two weight losses, the first one (1% weight loss) corresponding to the desorption of water molecules and the second one (2% weight loss) to the decomposition of the PAA molecules.

**Scheme 2:**
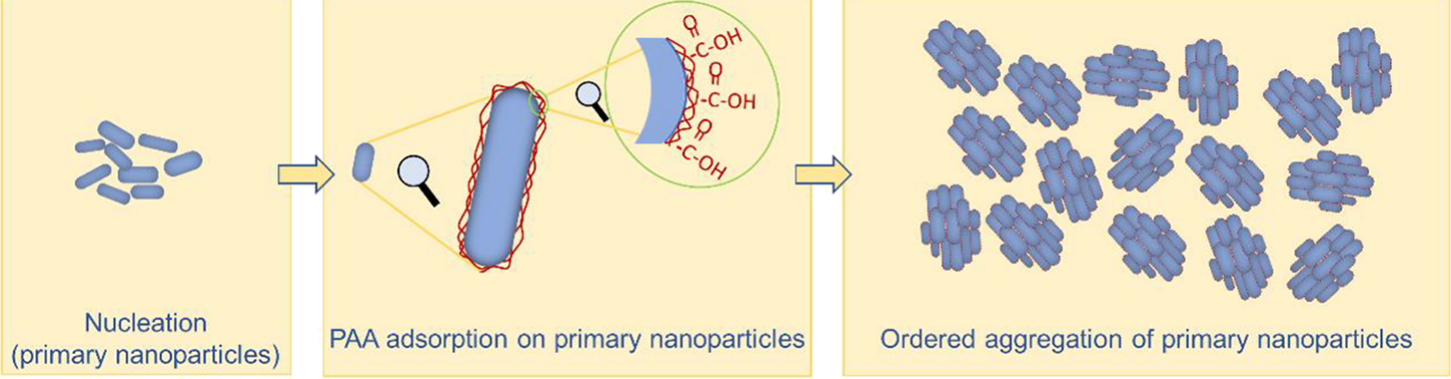
Schematic Representation of the Suggested Particle Formation Mechanism in the Presence of PAA

The incorporation of PAA molecules to the primary particles might be produced shortly after nucleation. The presence of PAA molecules on the primary particles’ surface must increase the repulsive forces among them as a result of both a higher negative surface charge (coming from the PAA carboxylate groups as PAA is deprotonated at basic pH values) and the effect of the steric hindrance.^36^ As a consequence, the force balance mentioned above is altered and so are the aggregation path and the morphological characteristics of the resulting aggregates. A schematic representation of the suggested particle formation mechanism in the presence of PAA is shown in Scheme 2.

The presence of PAA molecules on the NP surface is also beneficial for biosensing applications for two reasons: (i) they provide carboxylate groups that may favor colloidal stability under physiological conditions and (ii) the carboxylate groups work as anchor sites for the subsequent conjugation to proteins (e.g., monoclonal antibodies against IL-6). In summary, our developed one-pot hydrothermal reaction rendered uniform, water-dispersible, nanometer-size Zn_2_GeO_4_ particles functionalized with 2 weight % PAA.

This is, to the best of our knowledge, the only method reported until now that allows obtaining one-pot, surface-functionalized Zn_2_GeO_4_ NPs with a regular shape and a uniform size. In addition, it is important to note that, as far as we know, this is the first report of uniform Zn_2_GeO_4_ NPs with morphology different from rods. This is important for biomedical applications such as biosensing and bioimaging, because it has been shown that the shape of the NPs can have an important effect on their interaction with cells.^37−39^ Therefore, the availability of synthesis methods that make it possible to obtain Zn_2_GeO_4_ NPs with a shape different from that of rods, classically found in the literature, could be of great interest to optimize their potential application in biomedicine.

**Figure 4:**
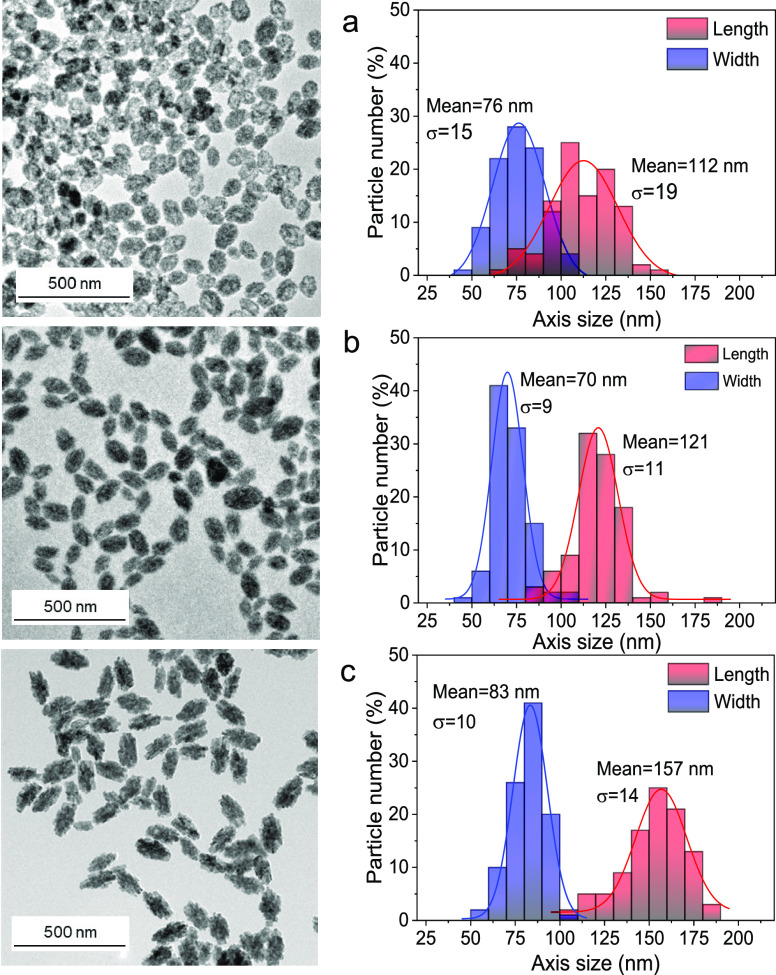
TEM micrographs and corresponding size histograms of Zn_2_GeO_4_ NPs synthesized under the experimental conditions described in Figure 1 but using (a) a CO as the heating source, (b) 100 °C as the reaction temperature, and (c) GeO_2_ and Zn(OAc)_2_ concentrations of 0.02 and 0.04 M, respectively.

Finally, it must be mentioned that the modification of other experimental parameters, with regard to those described in Figure 1, while keeping the rest constant, did not cause such a drastic modification in the morphology of the precipitated particles as it was the case for the absence of PAA described above. For example, very uniform, although longer and wider (∼76 nm × ∼112 nm) NPs, were obtained when the reaction was carried out in a conventional oven (CO) instead of a microwave oven (Figure 4a). Uniform NPs with a similar size to the latter (∼70 nm × ∼121 nm) were observed when the reaction was carried out at 100 °C (Figure 4b) and, finally, larger and more anisometric (∼83 nm × ∼157 nm), but also uniform particles were precipitated when using double reactants concentrations (Figure 4c). The DLS measurements recorded in their aqueous suspensions gave *z*-average sizes of the order of the mean TEM dimensions, which demonstrated that the NPs were well dispersed in distilled water (Figure S2). All three precipitates showed XRD patterns compatible with rhombohedral Zn_2_GeO_4_ (Figure S3). The width of the reflections was very similar in all three cases, indicating a similar size of the constituent crystallites. Therefore, the above differences in the NP size must be due to variations in the number of building blocks resulting from the alteration of the magnitude of the repulsive forces produced because of the different synthesis conditions. In particular, the observed size increase produced as decreasing temperature and increasing precursors concentration would agree with a lower repulsion that follows the expected decrease of surface potential as decreasing temperature and increasing ionic strength.^33^ The effect of the heating source (MW or CO) may be speculatively related to the temperature effect. Thus, the slower heat transfer involved in the CO procedure as compared with that associated with the MW oven implies that the nucleation, growth, and aggregation events may start before reaching the target temperature in the former case.

Because of the lower size of the NPs obtained in the experimental conditions of Figure 1 (with both length and width dimensions in the nanometer range) compared with those in Figure 4, we selected those NPs for further studies in this work.

### 3.2 Doping of Zn_2_GeO_4_ NPs with Mn^2+^

Due to their similar ionic radii (0.60 and 0.66 Å for Mn^2+^ and Zn^2+^, respectively, in IV coordination)^40^ and same oxidation state, Mn^2+^ ions are expected to readily substitute the Zn^2+^ ions in the Zn_2_GeO_4_ crystal structure. Mn^2+^-doped Zn_2_GeO_4_ NPs were prepared using the method described in Figure 1 for Zn_2_GeO_4_ NPs and adding manganese acetate to the starting solution. Different manganese acetate concentrations were used to synthesize Zn_2_GeO_4_ NPs with different Mn^2+^ doping levels (from 0.25 up to 3.00 mol % Mn^2+^ in Zn_2_GeO_4_). The resulting NPs presented the same shape (Figure S4), size (Figure S5), and dispersibility in distilled water (Figure S6) as the undoped ones. In all cases, ICP measurements (Table 1) indicated that around 70% of the nominal Mn^2+^ content was incorporated into the NPs. Therefore, the doping process did not alter the morphological characteristics of the resultant NPs. The crystal structure was not modified either as inferred from the XRD patterns of the Mn^2+^-doped samples (Figure S7), which were almost identical to one another and to that of the undoped sample. No reflection shift was observed among the patterns likely due not only to the similar ionic radii of Zn^2+^ and Mn^2+^ but also to the low Mn^2+^ doping level used.

**Table 1:** Nominal and Experimental (from ICP) Mn^2+^ Content of the Zn_2_GeO_4_:*x*%Mn^2+^ NPs (*x* = [Mn/(Mn + Ge)]*100)

nominal *x* (%)	experimental *x* (%)
0.25	0.16
0.50	0.32
1.00	0.66
2.00	1.22
2.50	1.86
3.00	2.34

### 3.3 Luminescence Properties

**Figure 5:**
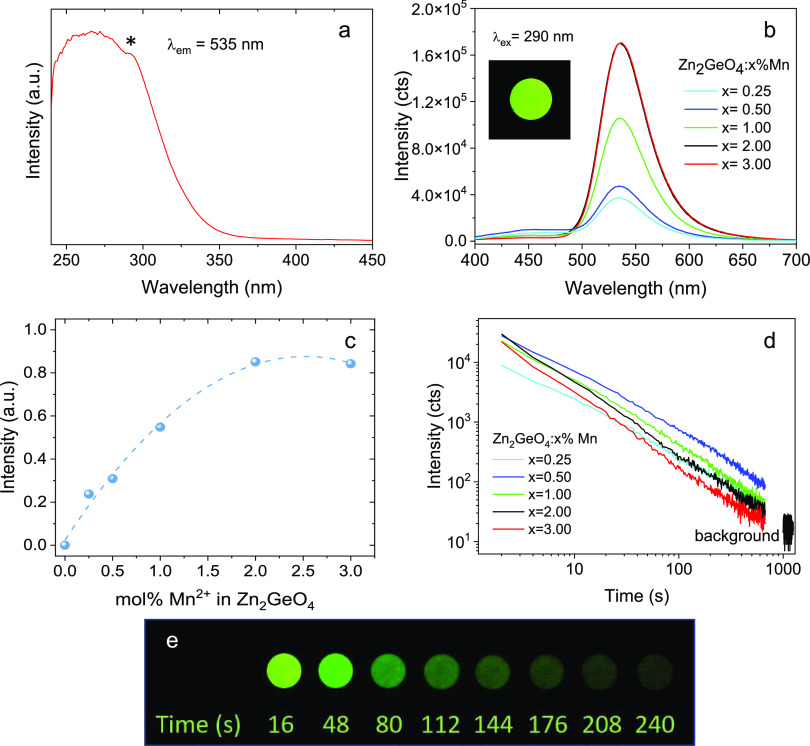
(a) Excitation spectrum of Zn_2_GeO_4_:0.50%Mn^2+^ NPs. The asterisk indicates the shouder at lower energies as described in the text. (b) Emission spectra of Zn_2_GeO_4_:Mn^2+^ NPs suspended in distilled water (1 mg·mL^–1^). The inset is a photograph of the Zn_2_GeO_4_:0.50%Mn^2+^ sample taken under UV light. (c) Integrated area under the curve of the emission spectra shown in b. (d) Persistent luminescence decays of Zn_2_GeO_4_:Mn^2+^ NPs suspended in distilled water (1 mg·mL^–1^) recorded after illumination with 290 nm light for 5 min. (e) Digital photographs at different time intervals of the Zn_2_GeO_4_:0.50% Mn^2+^ powder after irradiation at 312 nm for 5 min.

The excitation spectrum of Zn_2_GeO_4_:0.25%Mn^2+^ NPs (Figure 5a), recorded at an emission wavelength of 535 nm, consisted of a broad UV band with a shoulder at around 290 nm. The rest of the Mn^2+^-doped samples showed a very similar excitation spectrum to this one and are not shown for simplicity. The high-energy side of the excitation band can be ascribed to the transition from the valence band to the conduction band of the host Zn_2_GeO_4_ crystal followed by energy transfer to Mn^2+^.^41^ On the other hand, the shoulder at lower energies (marked with an asterisk) has been assigned to charge transfer from O^2–^ to Mn^2+^,^42^ although it has also been ascribed by other authors to the transition between the valence band and the impurity level of oxygen vacancies, followed by the energy transfer to Mn^2+^.^41^ The Zn_2_GeO_4_:Mn^2+^ NPs, excited under UV light, emitted green light (inset of Figure 5b), characteristic of Mn^2+^ ions located in tetrahedral sites, in agreement with the crystal structure of the phenakite Zn_2_GeO_4_ host, which provides only tetrahedral sites for substitution (as described in the introduction). Figure 5b shows the emission spectra of aqueous suspensions containing Zn_2_GeO_4_:*x*%Mn^2+^ NPs (*x* = 0.25% up to 3.00%) recorded under 290 nm excitation. For an accurate comparison, the NP concentration was kept the same in all suspensions (1 mg·mL^–1^). In agreement with the observed green luminescence, the emission spectra consisted essentially of a broad band centered at 535 nm, corresponding to the transition from the excited ^4^T_1g_ to the ground ^6^A_1g_ state of the Mn^2+^ ion,^43^ which indicates that the Mn ions keep their 2+ oxidation state after incorporation into the Zn_2_GeO_4_ matrix^44,45^ A low-intensity broad band centered at ∼450 nm could also be observed in the spectra of Figure 5b, especially in the low-doped samples, that has been assigned to the radiative recombination of electrons in the Zn_2_GeO_4_ matrix.^46^ It can be observed that, although the emission band at 535 nm did not shift in energy with Mn^2+^ doping level, the intensity of the emission increased with increasing Mn^2+^ content from *x* = 0.25% up to *x* = 2.00% while it did not increase further for the 3.00%Mn^2+^-doped sample due to the concentration quenching effect.^47^ The dependence of emission intensity with Mn^2+^ content can be clearly observed in Figure 5c, where the integrated area under the curve of the emission spectra, in the 400–700 nm interval, has been plotted versus the Mn^2+^ concentration. It can therefore be concluded that the optimal doping content for the highest photoluminescence emission in this system is between 2.00 and 3.00 mol % Mn^2+^. This interval is within the range found for other Zn_2_GeO_4_:Mn^2+^-based materials, which lie between 0.25 and 4.00%.^16,31,41,43,45,48−50^

Figure 5d shows the persistent luminescence decay curves, recorded at an emission wavelength of 535 nm after excitation at 290 nm for 5 min, of aqueous suspensions containing the same amount of Zn_2_GeO_4_:Mn^2+^ NPs (1 mg·mL^–1^) with different doping levels. It can be observed that in spite of the low concentration of NPs in the suspension, the green emission could be detected long after stopping the excitation for all suspensions. The afterglow time of all of them was well inside the time scale needed for the design of the interleukin-6 sandwich immunoassay, as will be shown below. It is remarkable that in spite of the low emission luminescence shown by the 0.50%-doped sample it showed the highest persistent luminescence at practically any time after stopping the excitation. The Mn^2+^ content that gave rise to the highest persistent luminescence was therefore different from that giving rise to the highest photoluminescence brightness (between 2.00 and 3.00% Mn^2+^). Such a difference can be explained by the different mechanisms involved in both processes. The latter consists of the simple excitation of the Mn^2+^ ions through energy transfer from the matrix followed by de-excitation to the Mn^2+^ ground state (^4^T_1g_→^6^A_1g_).^51^ Photoluminescence intensity is therefore directly linked to the number of emitting centers, so the highest emission is generally obtained for the highest doping content before concentration quenching occurs. In contrast to photoluminescence, persistent luminescence in Zn_2_GeO_4_:Mn^2+^ is a more complex process involving storage of the excitation energy by trapping charges (electrons and/or holes) in lattice defects (oxygen, zinc, and germanium vacancies and interstitial Zn) followed by charge release, recombination, and light emission from the luminescence center (Mn^2+^).^8^ The increase of Mn^2+^ content could somehow affect the distribution and depth of trapping centers so that Mn^2+^ contents higher than 0.50% lead to a decrease in the intensity of persistent luminescence. In fact, the plot of the normalized persistent luminescence decays (Figure S8a) reveals that increasing Mn^2+^ content produces a faster luminescence decay rate, although a deeper analysis of the persistent luminescence mechanism, involving distribution and depth of the traps, is out of the scope of this study.

Although the optimal Mn^2+^ content for the photoluminescence of Zn_2_GeO_4_:Mn^2+^ samples has been searched by several authors, as described above, this is not the case for the optimal Mn^2+^ content for persistent luminescence, which, to the best of our knowledge, has not been optimized in the literature before. Digital photos of the Zn_2_GeO_4_:0.50%Mn^2+^ powder at different decay time intervals are shown in Figure 5e for direct observation of the persistent luminescence. The photographs corresponding to NPs with other Mn^2+^ doping levels (from 0.25 up to 3.00 mol %) are shown in Figure S8b. They show persistent luminescence that agrees well with the decay curves of Figure 5d.

### 3.4 Stability of the Nanoparticles in Phosphate Buffer Saline

The colloidal, chemical, and photostability of the NPs was examined in PBS suspensions at pH = 6.0 and 7.4, which are experimental conditions at which the NPs are submitted during the immunoassay described in Section 3.5.

#### 3.4.1 Colloidal and Chemical Stability

**Figure 6:**
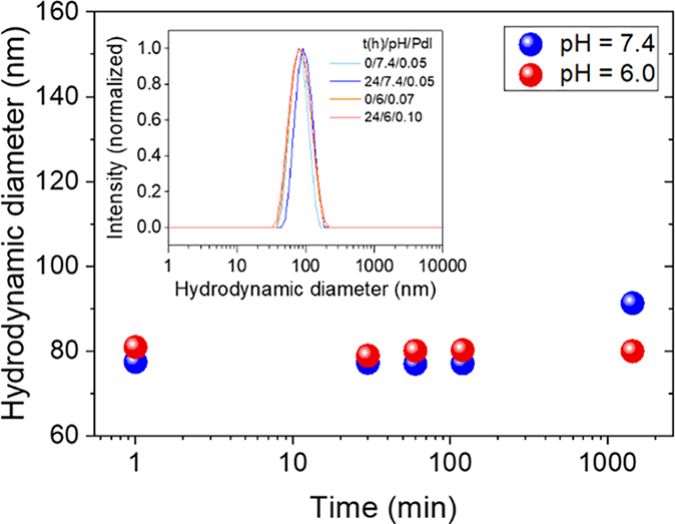
Z-average size of Zn_2_GeO_4_:0.50%Mn^2+^ NPs dispersed in PBS at pH = 6.0 and pH = 7.4 as a function of time. The inset shows the intensity PSDs obtained from DLS at 0 and 24 h as well as the corresponding PdI values.

Figure 6 shows the *z*-average size values obtained from DLS recorded at different time intervals (up to 90 h) in PBS suspensions of Zn_2_GeO_4_:0.50%Mn^2+^ NPs (0.25 mg·mL^–1^) at pH = 6.0 and pH = 7.4. Both suspensions were kept at room temperature, without any stirring or shaking, during the whole measuring period. All recorded values, except those obtained after 90 h, were well below 100 nm and the width of their intensity PSDs, given by the PdI values, did not appreciably change with time (inset of Figure 6). This result demonstrates that the NPs were colloidally stable in PBS at both pH values for, at least, 24 h. Their high colloidal stability can be ascribed to both electrostatic and steric interactions due to the presence of PAA chains at the surface of the NPs,^36^ as explained before.

**Figure 7:**
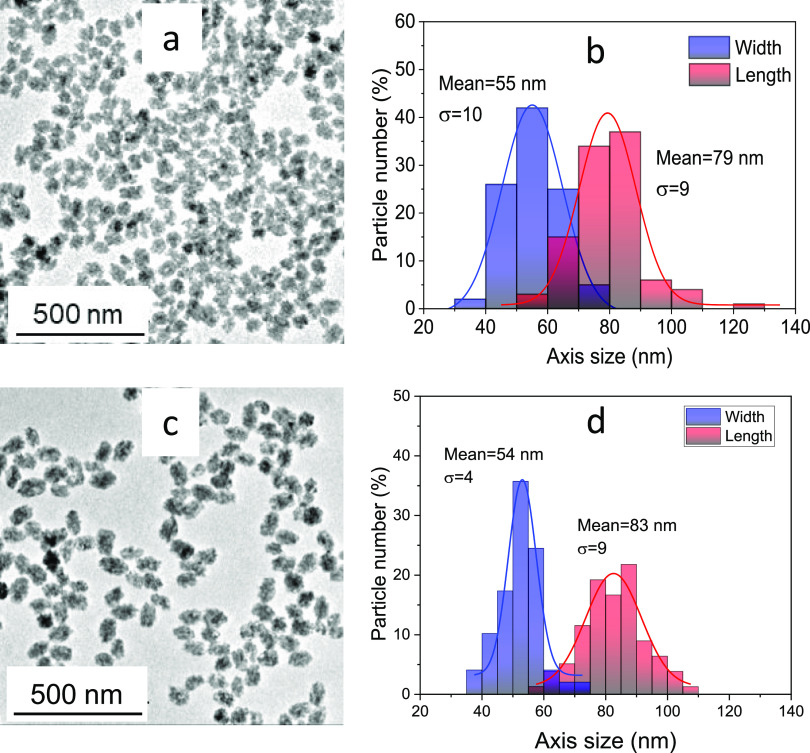
TEM micrographs (a and c) and size distribution histograms (b and d) of Zn_2_GeO_4_:0.50%Mn^2+^ NPs dispersed in PBS at pH = 7.4 (a and b) and pH = 6.0 (c and d) after 1 week.

On the other hand, the chemical stability of the Zn_2_GeO_4_:0.50%Mn^2+^ NPs was evaluated through TEM and ICP analyses after 1 week in a PBS dispersion at pH = 7.4 and pH = 6.0. The TEM micrographs (Figure 7a,c) and the size histograms (Figure 7b,d) showed that neither the shape nor the size of the NPs changed after this time at any pH value. Likewise, the ICP analyses indicated that only 0.3 and 0.9% of Zn was dissolved pH =7.4 and at pH = 6.0, respectively after 1 week as compared with the total Zn amount contained in the NPs. This result demonstrates the high chemical stability of the NPs in PBS medium at both pH values and warrants their stability during the immunoassay.

#### 3.4.2 Photostability

**Figure 8:**
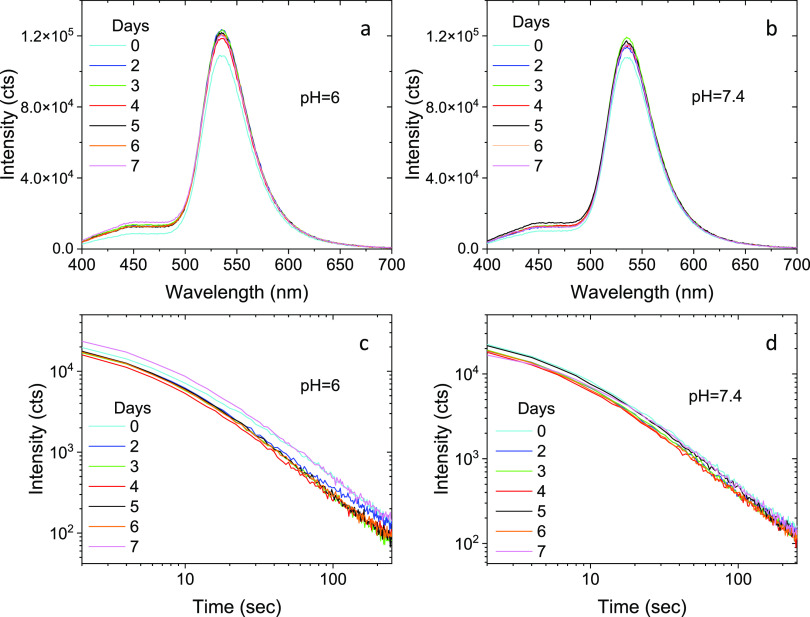
Emission spectra (a and b) and persistent luminescence decay curves (c and d) of Zn_2_GeO_4_:0.50%Mn^2+^ NPs dispersed in PBS, at pH = 6.0 and pH = 7.4, for several periods of time up to 1 week.

The photostability of the Zn_2_GeO_4_:0.50%Mn^2+^ NPs suspended in PBS at pH = 6.0 and pH = 7.4 was tested by recording the emission spectra and the persistent luminescence decay curves of the suspensions (1 mg·mL^–1^) for 1 week. As observed in Figure 8, no significant change was produced in the intensity of the emission during excitation (emission spectra in Figure 8a,b) or in the duration of the luminescence after switching off the UV excitation source (Figure 8c,d) at any pH value. This result indicates a good photostability of the NPs in PBS at pH = 6.0 and 7.4 during, at least, 1 week.

### 3.5 Application of Zn_2_GeO_4_:0.50%Mn^2+^ PLNPs in the Detection of IL-6

Owing to their excellent persistent luminescence and to their carboxylate-functionalized surface, we explored the application of Zn_2_GeO_4_:0.50%Mn^2+^ PLNPs as the signal transducer element for the design and fabrication of an autofluorescence-free immunoassay for the detection of IL-6. The IL-6 immunoassay is based on the formation of the (Ab_*c*_) – (IL-6) – (Ab_*d*_-PLNPs) sandwich, where Ab_*c*_ and Ab_*d*_ are the capture and detection antibodies, respectively (see Scheme 1). While the capture antibodies (Ab_*c*_) were anchored to the well plate, the PLNPs were conjugated to detection antibodies (Ab_*d*_) by carbodiimide coupling chemistry.^52,53^ The Ab_*c*_ and PLNPs-Ab_*d*_ concentrations were chosen according to a 2D assay (Figure S9). After immobilizing the capture antibodies to the surface of the wells, BSA (0.1%) was incubating as the blocking agent, and then undiluted human serum, undiluted human plasma, or PBS (pH 7.4), spiked with known concentrations (0–10^6^ pg·mL^–1^) of IL-6, was added. After the specific capture of IL-6, the PLNPs-Ab_*d*_ nanoprobes were also added which allowed the formation of the sandwich immunoassay. After rinsing, the persistent luminescence emitted by the NPs was recorded after UV excitation. The signal intensity was proportional to the number of PLNP nanoprobes which was in turn proportional to the concentration of IL-6 in the sample. In case IL-6 was not present in the sample, the sandwich assay could not be formed and therefore the PLNPs-Ab_*d*_ nanoprobes would not bind and luminescence would not be observed.

**Figure 9:**
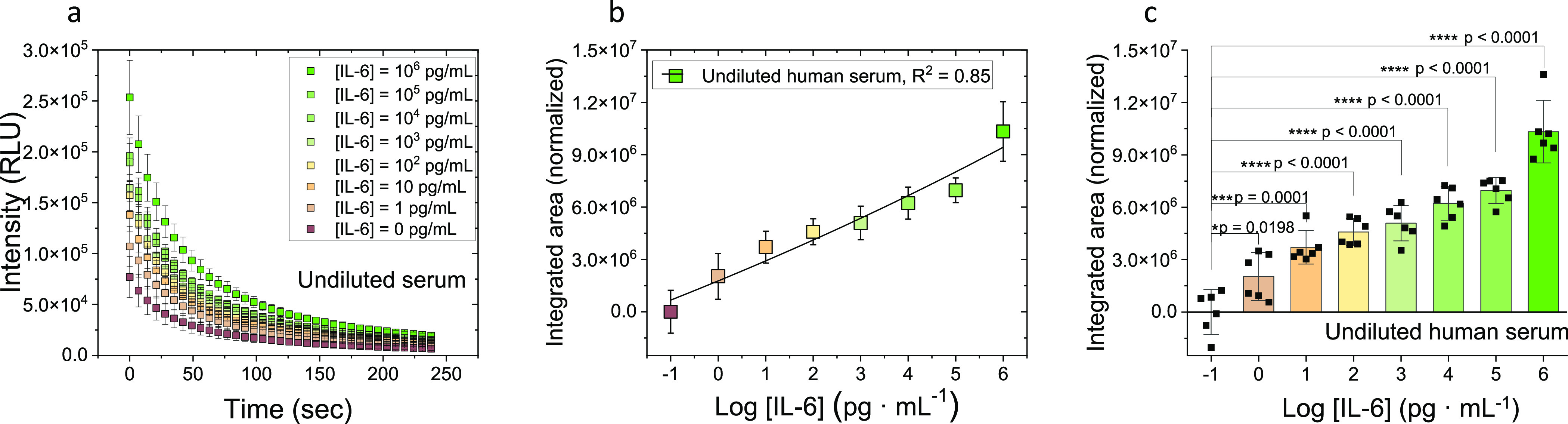
IL-6 sandwich immunoassay in undiluted human serum ([Ab_*c*_] = 10 μg·mL^–1^, [Ab_*d*_] = 2.36 μg·mL^–1^, *n* = 6). (a) Persistent luminescence decays. RLU = Relative luminescence units. (b) Normalized integrated area under the decay curves shown in Figure 9a versus IL-6 concentration. (c) Unpaired and two-tailed t-test obtained from the data analysis of Figure 9b.

The persistent luminescence decay curves of the sandwich immunoassay were recorded in the presence of different concentrations of IL-6 (from 0 to 10^6^ pg·mL^–1^, *n* = 6) spiked in undiluted serum (Figure 9a), in undiluted plasma (Figure S10a) and in PBS (pH = 7.4) (Figure S10d), after 150 s UV excitation. The integrated area below the decay curves, in the 0–240 s interval, was then plotted vs IL-6 concentration to construct the calibration curves in undiluted human serum (Figure 9b), in undiluted human plasma (Figure S10b), and in PBS (pH = 7.4) (Figure S10e). In the case of IL-6 spiked in undiluted human serum samples, the unpaired and two-tailed t-test analyses (95% confidence level) showed significant differences, with respect to the negative control, from IL-6 concentrations as low as 1 pg·mL^–1^, and for a wide working range (Figure 9c). The t-test analyses for IL-6 spiked in undiluted human plasma and PBS (pH = 7.4) samples can be found in the Supporting information (Figure S10c,f, respectively). In conclusion, it has been proven that our PLNPs can be used as nanoprobes for the detection of IL-6 in clinical applications such as the sepsis, where healthy adults without inflammation have low IL-6 concentrations (<10 pg·mL^–1^)^54,55^ compared to septic episodes where the levels of this cytokine can dramatically increase.^54,56^ However, it should be noticed that the negative control ([IL-6] = 0 pg·mL^–1^) showed some signal, which is likely due to some nonspecific absorption of the PLNPs-Ab_*d*_ nanoprobes. Therefore, more efforts should be made to improve the assay sensitivity through the minimization of nonspecific absorption.

## 4 Conclusions

Uniform Zn_2_GeO_4_:Mn^2+^ NPs with an ellipsoidal shape (85 nm × 60 nm) and functionalized with PAA were synthesized by a one-pot hydrothermal reaction. PAA not only acted as the functionalizing agent but it was an indispensable reagent to obtain uniform NPs through the ordered aggregation of the building blocks. PAA might also be responsible for the high colloidal stability of the NPs dispersed in water through electrostatic and steric interactions. The Zn_2_GeO_4_:Mn^2+^ NPs emitted green light under UV excitation, the maximum emission intensity being obtained for the Zn_2_GeO_4_ NPs doped with 2.50% Mn^2+^. The green emission persisted well after switching off the UV excitation, with the 0.50%Mn^2+^-doped NPs showing the longest persistence. The potential biosensing application of these one-pot functionalized, persistent luminescence NPs was demonstrated with the fabrication of an autofluorescence-free persistent luminescence sandwich immunoassay to detect interleukin-6 in human specimens (undiluted serum and plasma). The fabricated immunoassay was able to detect as low as 1 pg·mL^–1^ of IL-6 spiked in undiluted human serum samples.

## Supporting Information Available

TEM micrographs and DLS curves of NPs obtained in the same experimental conditions as those described in Figure 1 of the article but using different PAA concentrations, DLS curves and XRD patterns of Zn_2_GeO_4_:Mn^2+^ NPs synthesized in different experimental conditions; TEM, size histograms, DLS curves, and XRD patterns of Zn_2_GeO_4_ doped with different Mn^2+^ contents, normalized persistent luminescence decays and digital photographs of Zn_2_GeO_4_:Mn^2+^ NPs with different Mn^2+^ contents after excitation for 5 min, additional experimental details including a 2D-assay to select the Ab_*c*_ and Ab_*d*_-PLNPs concentrations, and IL-6 sandwich immunoassay in undiluted human plasma and PBS (pH = 7.4) (PDF)
