# Supplementary material for: Persistent Luminescence Zn2GeO4:Mn2+ Nanoparticles Functionalized with Polyacrylic Acid: One-Pot Synthesis and Biosensing Applications
Source: ACS Appl Mater Interfaces. 2023 Mar 27;15(17):20613–24. doi: 10.1021/acsami.2c21735 (PMC10165609; doi:10.1021/acsami.2c21735)
Supplement: Supplementary file 1 — am2c21735_si_001.pdf [file am2c21735_si_001.pdf]

Supporting information

Persistent Luminescence $Zn_2GeO_4:Mn^{2+}$ Nanoparticles Functionalized with Polyacrylic Acid: One-pot Synthesis and Biosensing Applications

Roxana M. Calderón-Olvera,[1†] Encarnación Arroyo,[1†] Aaron M. Jankelow[2,3], Rashid Bashir[2,3,4,5,6] Enrique Valera[2,3], Manuel Ocaña,[1*] Ana Isabel Becerro[1*]

[1]Instituto de Ciencia de Materiales de Sevilla (CSIC-US), c/Américo Vespucio, 49, 41092 Seville, Spain.

[2]Department of Bioengineering, University of Illinois at Urbana-Champaign, Urbana, IL 61801, USA.

[3]Nick Holonyak Jr Micro and Nanotechnology Lab, University of Illinois at Urbana-Champaign, Urbana, IL 61801, USA.

[4]Department of Electrical and Computer Engineering, University of Illinois at Urbana-Champaign, Urbana, IL 61801, USA.

[5]Department of Mechanical Science and Engineering, University of Illinois at Urbana-Champaign, Urbana, IL 61801, USA.

[6]Center for Genomic Diagnostics, Woese Institute for Genomic Biology, Urbana, IL 61801, USA.

Keywords: persistent luminescence, nanoparticles, zinc germanate, functionalization, colloidal stability, photostability, biosensing, immunoassay

[†]These authors had equal contributions
[*]Corresponding authors: mjurado@icmse.csic.es and anieto@icmse.csic.es

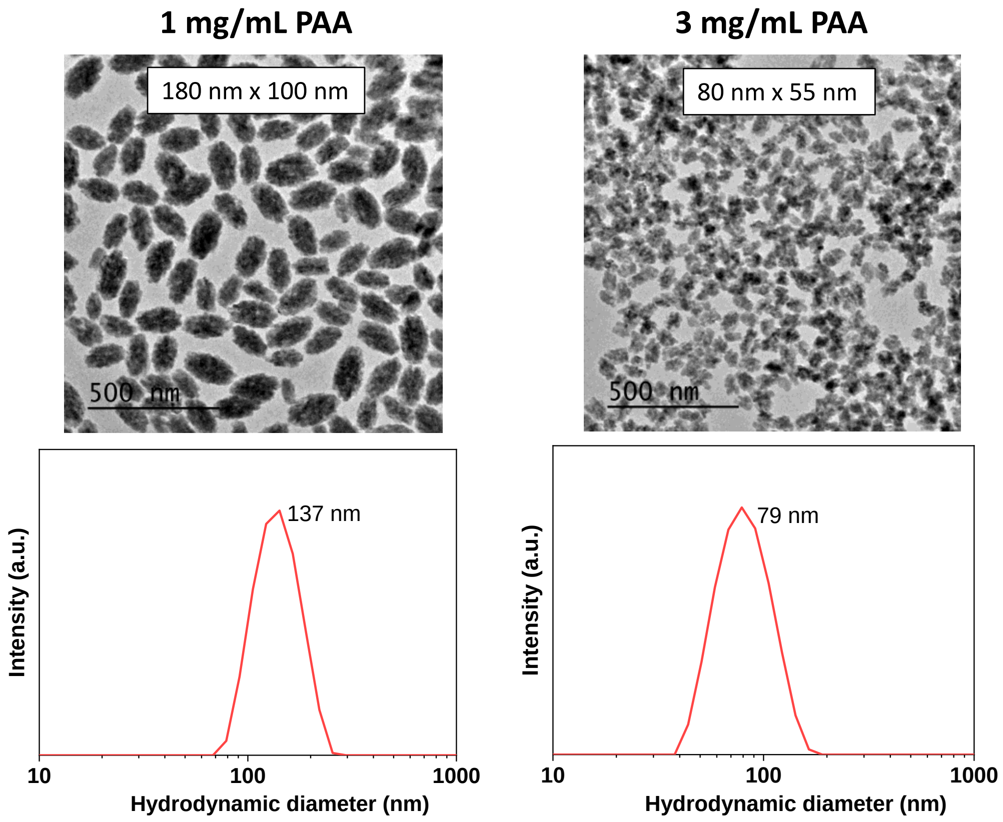

Figure S1. TEM micrographs and DLS curves of NPs obtained in the same experimental conditions as those described in Figure 1 of the article but using different PAA concentrations. The mean length and width dimensions of the NPs are shown at the top of the micrographs.

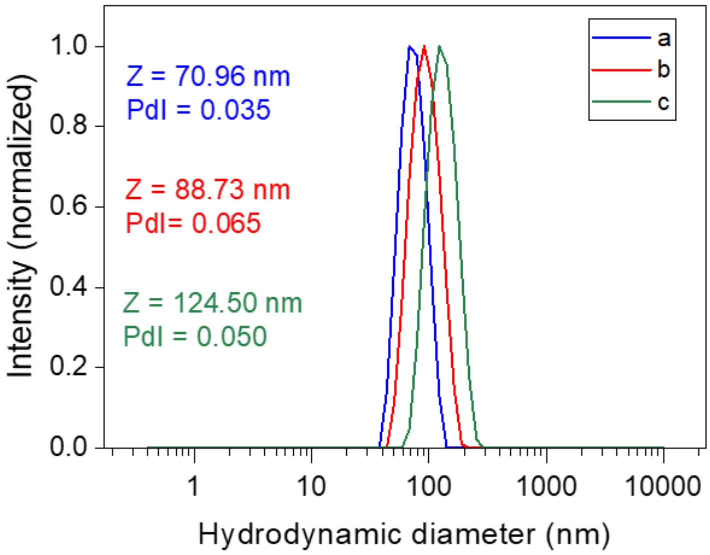

Figure S2. Intensity PSDs obtained from DLS (in distilled water) corresponding to the Zn_2GeO_4 particles shown in Figure 4 of the article.

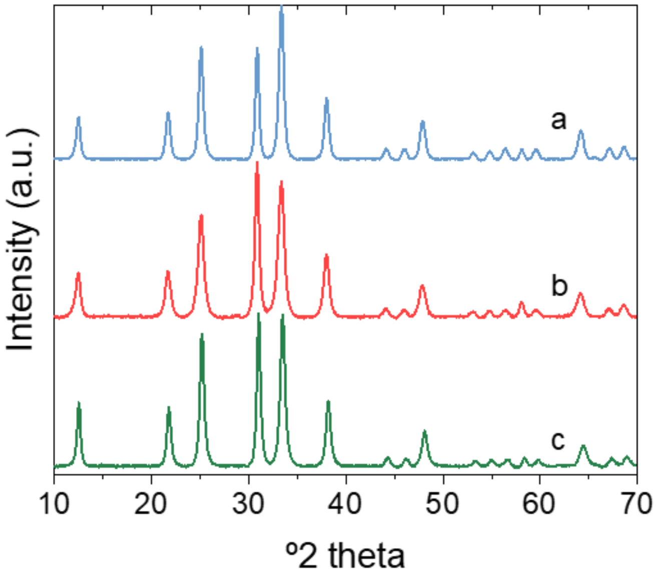

Figure S3: X-ray diffraction patterns corresponding to the Zn_2GeO_4 particles shown in Figure 4 of the article.

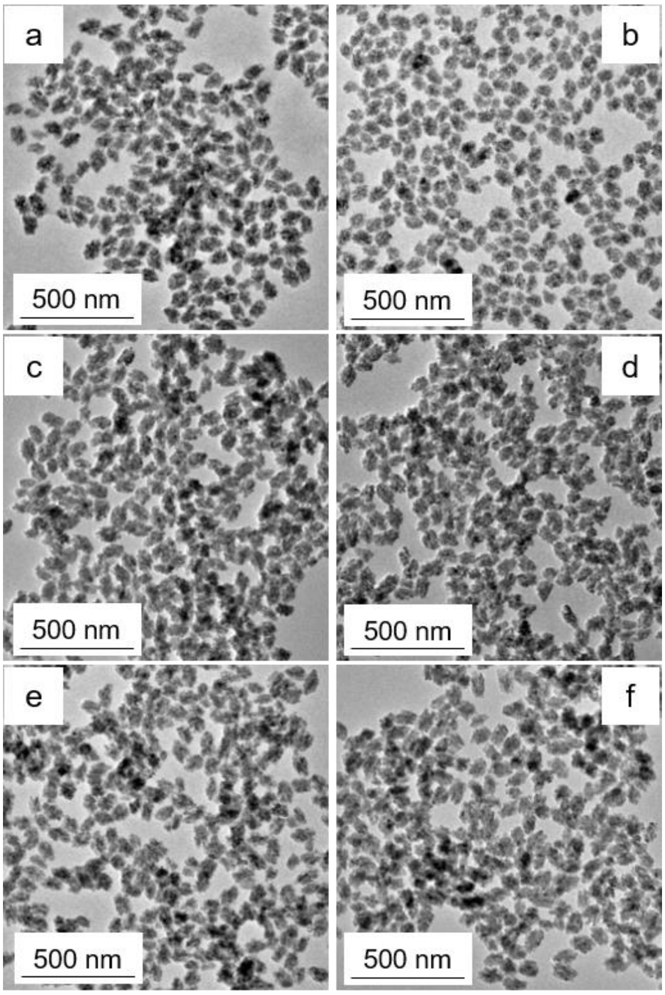

Figure S4: TEM micrographs corresponding to Zn_2GeO_4 NPs doped with different nominal Mn^{2+} contents: a) 0.25% Mn^{2+}, b) 0.50% Mn^{2+}, c) 1.00% Mn^{2+}, d) 2.00% Mn^{2+}, e) 2.50% Mn^{2+}, and f) 3.00% Mn^{2+}.

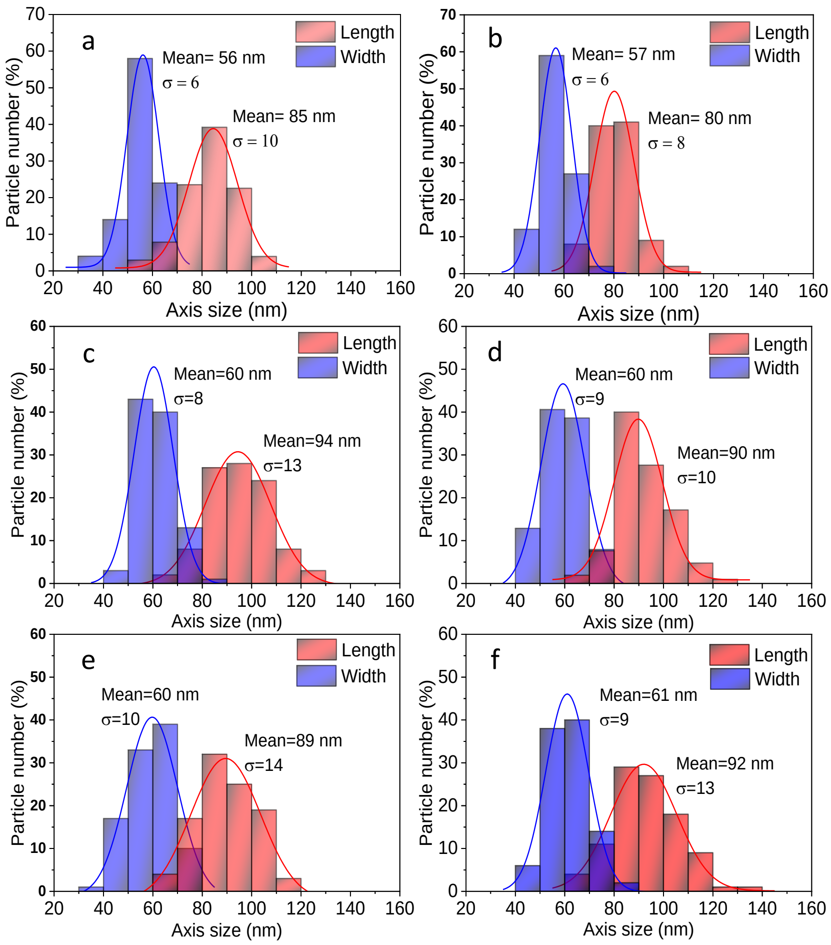

Figure S5: Size distribution histograms obtained from the TEM micrographs of Figure S4 corresponding to Zn_2GeO_4 NPs doped with different nominal Mn^{2+} contents: a) 0.25% Mn^{2+}, b) 0.50% Mn^{2+}, c) 1.00% Mn^{2+}, d) 2.00% Mn^{2+}, e) 2.50% Mn^{2+}, and f) 3.00% Mn^{2+}.

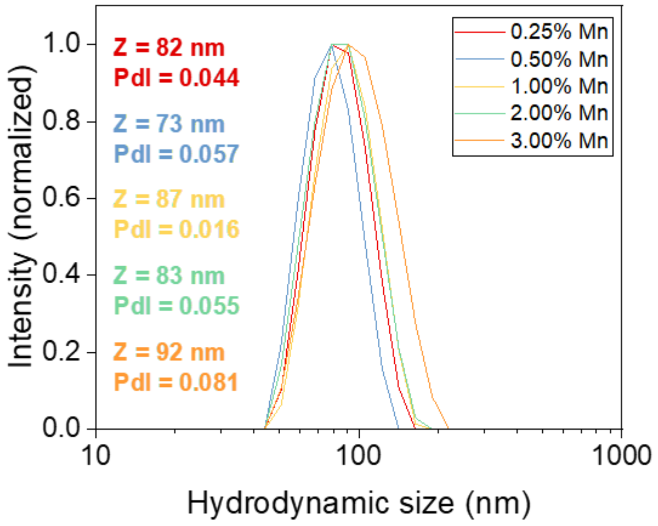

Figure S6: Intensity PSDs obtained from DLS (in distilled water) of the NPs shown in Figure S4.

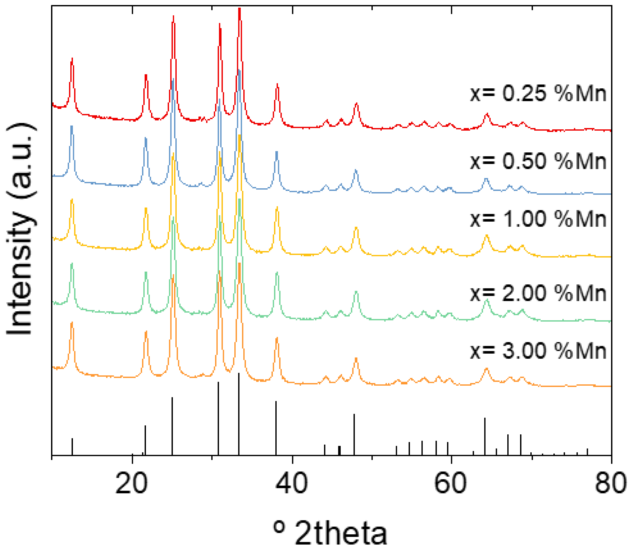

Figure S7: XRD patterns of the NPs shown in Figure S4. Bottom lines correspond to PDF 00-011-0687 (rhombohedral Zn_2GeO_4).

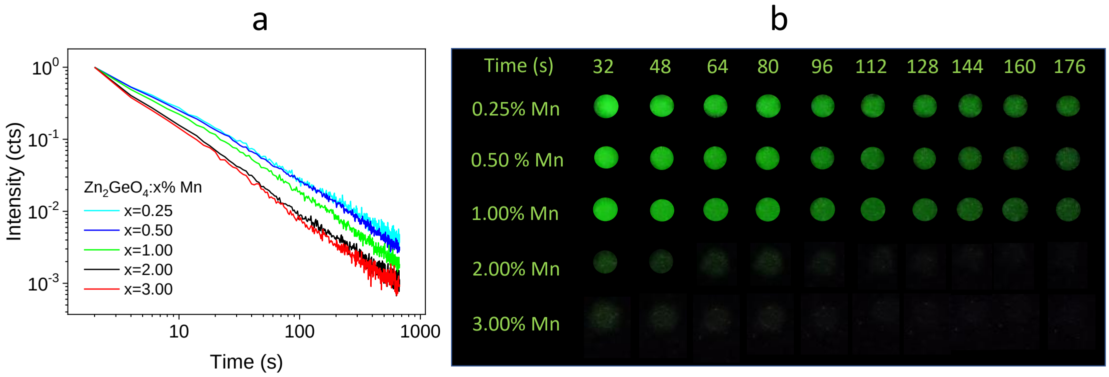

Figure S8: a) Normalized persistent luminescence decays of $Zn_2GeO_4:Mn^{2+}$ NPs suspended in water (1 mg·mL^{-1}) recorded after illumination with 290 nm light for 5 minutes. b) Digital photographs at different time intervals of the Zn_2GeO_4 NPs doped with different Mn^{2+} contents after photoexcitation for 5 minutes with UV lamp (312 nm).

2D-assay to select the Ab_c and PLNPs-Ab_d concentrations

Following the procedure explained in the main text for the sandwich immunoassay, different concentrations of Ab_c (21.21, 10.60, 5.30, and, 0 µg·mL^{-1}) and, Ab_d (2.36, 1.18, and, 0 µg·mL^{-1}) were used. The persistent luminescence decays of suspensions containing IL-6 (10^6 pg·mL^{-1}) and the different concentrations of PLNPs-Ab_d and Ab_c were recorded after excitation with UV light (312 nm, 90 s). The integrated area of the persistent luminescence decays was plotted versus the Ab_c concentration for each Ab_d concentration (Figure S9). Considering a compromise between a high luminescence intensity and a non-saturated signal, the most suitable pair of concentrations of PLNPs-Ab_d attached to IL-6 and Ab_c linked to the well-plate was found to be 10 µg·mL^{-1} Ab_c and 2.36 µg·mL^{-1} Ab_d.

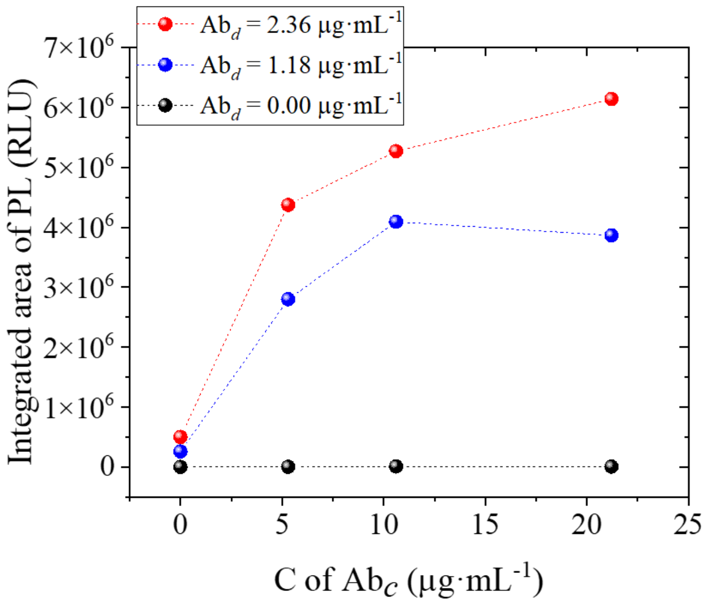

Figure S9: Integrated area of the persistent luminescence decays of suspensions containing IL-6 (10^6 pg·mL^{-1}) and different concentrations of PLNPs-Ab_d and Ab_c. Ab_d stands for detection antibodies and Ab_c for capture antibodies. RLU = Relative Luminescence Units.

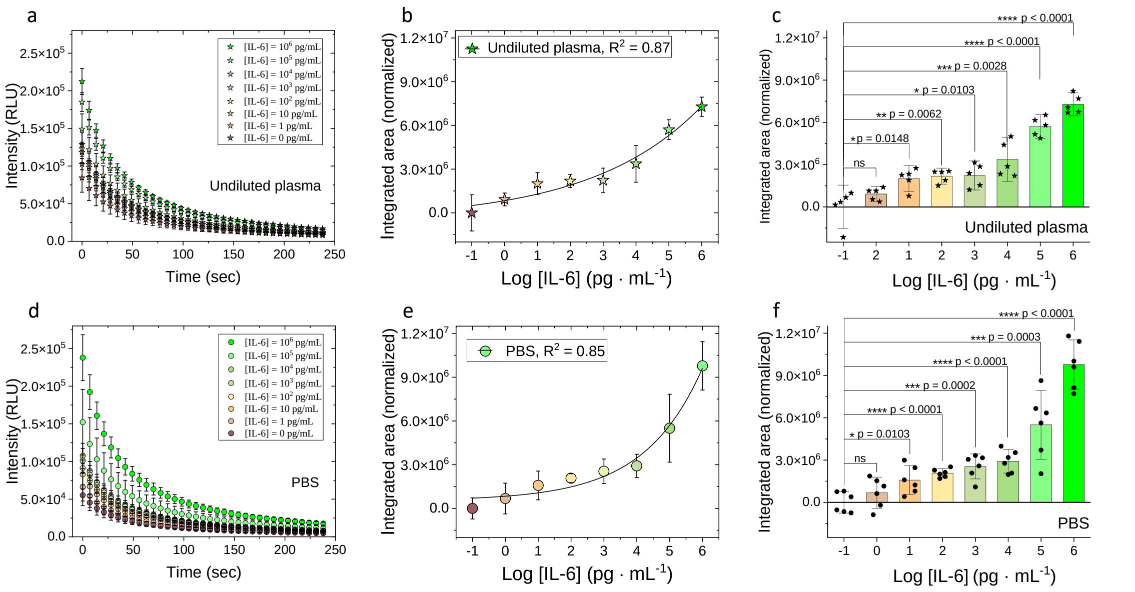

Figure S10: a-c) IL-6 sandwich immunoassay in undiluted human plasma ([Ab$_c$] = 10 µg·mL^{-1}, [Ab$_d$] = 2.36 µg·mL^{-1}, n = 5). a) Persistent luminescence decays. b) Normalized integrated area under the decay curves shown in Fig. S10a versus IL-6 concentration. c) Unpaired and two-tailed t-test obtained from the data analysis of Figure S10b. d-f) IL-6 sandwich immunoassay in PBS (pH = 7.4) ([Ab$_c$] = 10 µg·mL^{-1}, [Ab$_d$] = 2.36 µg·mL^{-1}, n = 6). d) Persistent luminescence decays. e) Normalized integrated area under the decay curves shown in Fig. S10d versus IL-6 concentration. f) Unpaired and two-tailed t-test obtained from the data analysis of Figure S10e. In both cases, the t-test analysis (95% confidence level) showed significant differences, with respect to the negative control, from IL-6 concentrations as low as 10 pg mL^{-1}, and for a wide working range.
